# The *ZNF76* rs10947540 polymorphism associated with systemic lupus erythematosus risk in Chinese populations

**DOI:** 10.1038/s41598-021-84236-3

**Published:** 2021-03-04

**Authors:** Yuan-yuan Qi, Yan Cui, Hui Lang, Ya-ling Zhai, Xiao-xue Zhang, Xiao-yang Wang, Xin-ran Liu, Ya-fei Zhao, Xiang-hui Ning, Zhan-zheng Zhao

**Affiliations:** 1 Nephrology Hospital, The First Affiliated Hospital of Zhengzhou University, Institute of Nephrology, Zhengzhou University, No.1, Jianshe Road, Erqi District, Zhengzhou, 4500052 Henan People’s Republic of China; 2 School of Pharmaceutical Sciences, Zhengzhou University, Zhengzhou, 450001 People’s Republic of China; 3 Department of Urology, The First Affiliated Hospital of Zhengzhou University, Zhengzhou, 4500052 Henan People’s Republic of China

**Keywords:** Immunology, Systemic lupus erythematosus

## Abstract

Systemic lupus erythematosus (SLE) is a typical autoimmune disease with a strong genetic disposition. Genetic studies have revealed that single-nucleotide polymorphisms (SNPs) in zinc finger protein (ZNF)-coding genes are associated with susceptibility to autoimmune diseases, including SLE. The objective of the current study was to evaluate the correlation between *ZNF76* gene polymorphisms and SLE risk in Chinese populations. A total of 2801 individuals (1493 cases and 1308 controls) of Chinese Han origin were included in this two-stage genetic association study. The expression of *ZNF76* was evaluated, and integrated bioinformatic analysis was also conducted. The results showed that 28 SNPs were associated with SLE susceptibility in the GWAS cohort, and the association of rs10947540 was successfully replicated in the independent replication cohort (*P*_replication_ = 1.60 × 10^−2^, OR 1.19, 95% CI 1.03–1.37). After meta-analysis, the association between rs10947540 and SLE was pronounced (*P*_*meta*_ = 9.62 × 10^−6^, OR 1.29, 95% CI 1.15–1.44). Stratified analysis suggested that *ZNF76* rs10947540 C carriers were more likely to develop relatively high levels of serum creatinine (Scr) than noncarriers (CC + CT vs. TT, *p* = 9.94 × 10^−4^). The bioinformatic analysis revealed that *ZNF76* rs10947540 was annotated as an eQTL and that rs10947540 was correlated with decreased expression of *ZNF76*. Remarkably, significantly reduced expression of *ZNF76* was confirmed by expression data from both our laboratory and an array-based expression database. Taken together, these results suggest that *ZNF76* rs10947540 is a possible susceptibility factor associated with SLE susceptibility. The mechanism underlying the relationship between *ZNF76* and SLE pathogenesis still requires further investigation.

## Introduction

Systemic lupus erythematosus (SLE) is a typical autoimmune disease that is characterized by increased generation of apoptotic debris and the presence of autoantibodies specific for nuclear components. Currently, the aetiology of SLE is not fully understood. It has been well documented that genetic factors are important for SLE predisposition.

Zinc finger proteins are DNA-binding proteins that control the transcription of a number of genes. Genetic studies have reported that single-nucleotide polymorphisms in zinc finger protein-coding genes are associated with susceptibility to autoimmune diseases, including SNPs in Eos (also known as Ikaros family zinc finger 4; *IKZF4*) being associated with alopecia areata^1^, those in zinc finger 432 (*ZNF432*) with asthma^2^, those in zinc finger 193 (*ZNF 193*) with rheumatoid arthritis^3^, those in zinc finger 365 (*ZNF365*) isoform D with Crohn's disease^4^, and those in zinc finger 3 (*IKZF3*) with Graves’ disease^5^. In a large-scale multiracial replication study, rs1453560 located between *IKAROS* family of zinc finger 3 (*AIOLOS; IKZF3*) and zona pellucida binding protein 2 (*ZPBP2*) was identified as a susceptibility locus for systemic lupus erythematosus^6^. Subsequently, the genetic association between rs907091 in the *IKZF3* gene and SLE was validated in a Chinese Han population^7^_,_ and the association between the *IKZF1* 5′ UTR variant rs1456896 and lupus nephritis in a northern Han Chinese population was also revealed^8^.

*ZNF76* is a novel transcriptional repressor targeting TATA-binding protein that has been shown to have an inhibitory effect on p53 activity by reporter assays and on endogenous target gene expression^9^. Integrated analysis of three original datasets, GSE72509, GSE20864, and GSE39088, from the Gene Expression Omnibus (GEO) database identified that the p53 signalling pathway may be implicated in SLE pathogenesis^10^. Considering both genetic clues concerning variants in zinc finger protein-coding genes contributing to susceptibility to autoimmune diseases, including SLE, and the biological functions of *ZNF76*, we aimed to explore the role of *ZNF76* in the pathogenesis of SLE.

Based on a previous SLE GWAS, we first replicated the tag SNP in our cohort to confirm the genetic association between *ZNF76* variants and SLE susceptibility. By using a public database, we explored the functional role of *ZNF76* rs10947540 and the differential expression of *ZNF76,* which was also validated by expression data from our centre.

## Methods

### Study populations

The replication cohort contained 1003 SLE patients and 815 healthy controls from the Henan population in the middle of China. All the patients were diagnosed by at least two experienced physicians according to the revised criteria for the classification of SLE from the American College of Rheumatology (ACR)^11^. Clinical data were retrospectively collected at the time of diagnosis. Written informed consent was obtained from each participant. This investigation was conducted according to the Declaration of Helsinki. The study was approved by the Medical Ethics Committee of Zhengzhou University First Hospital (2019-KY-134).

### SNP selection and genotyping

We focused on the 10-kb upstream and downstream regions of the *ZNF76* gene ranging from 35,217,512—35,273,762 on chromosome 6. Thirty-seven SNPs were covered by the ImmunoChip used in the GWAS and are listed in supplementary Table 1. Genetic association results were obtained from a previous publication^12^.

**Table 1:** Association of *ZNF76* polymorphisms with systemic lupus erythematosus susceptibility.

SNP	Minor allele	GWAS cohort (SLE vs. control, 490/493)			Replication population (SLE vs. control, 1003/815)			Meta-analysis	
		MAF (%)	*P*	OR (95%CI)	MAF (%)	*P*	OR (95%CI)	*P*	OR (95%CI)
rs10947540	C	39.6/30.2	1.3 × 10^−5^	1.51 (1.25–1.82)	33.6/29.9	1.6 × 10^−2^	1.19 (1.03–1.37)	9.62 × 10^−6^	1.29 (1.15–1.44)

*SNP* Single nucleotide polymorphism, *MAF* minor allele frequency, *OR* odds ratio, *CI* confidence interval.

**Figure 1:**
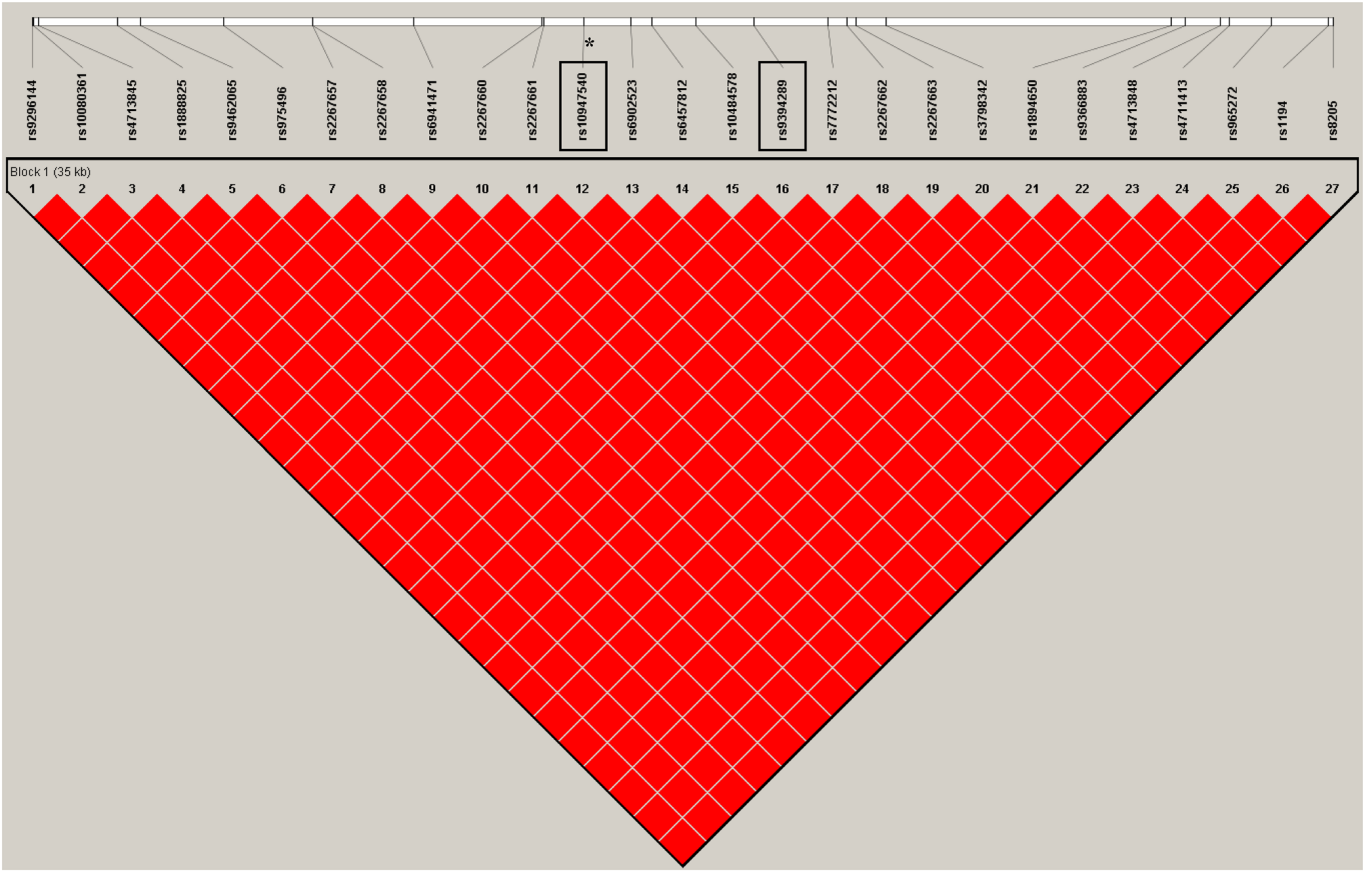
The LD maps of the 28 identified SLE-associated SNPs in 103 Chinese Han Beijing individuals according to 1000 genome project. The degrees of LD were estimated by CI method using Haploview4.2 (Cambridge, MA, USA) and a standard color scheme (D’/LOD) is used to display the LD pattern. Top signals (*p* = 1.31 × 10^−5^), rs10947540, rs9394289, rs2267663, rs1894650, rs9366883 were highlighted by black boxes and were in high linkage (D’ = 1.0, r-square = 1.0). The variant, rs10947540, further replicated in independent cohort was marked with asterisk.

A total of 28 SNPs associated with SLE susceptibility were identified in the GWAS cohort (supplementary Table 1)^12^. Five intronic polymorphisms within *ZNF76*, rs10947540, rs9394289, rs2267663, rs1894650, and rs9366883, were the top signals (*p* = 1.31 × 10^−5^) and were highly linked (D’ = 1.0, r-square = 1.0, Fig. 1) after analysing genotype data from 103 Chinese Han Beijing (CHB) individuals from the 1000 Genomes Project (Fig. 1). Further, rs10947540 was chosen as the tag SNP and was validated in the replication cohort. The Sequenom MassARRAY platform (Sequenom, Inc., San Diego, California, USA) was used for genotyping the replication cohort, and the genotyping yield was 99.5%.

Rigorous quality control of the GWAS cohort was performed previously and provided in a previous publication^12^. For the replication cohort, the missing genotyping rate of rs10947540 was 0.50% in the cases vs. 0.37% in the controls, and the Hardy–Weinberg equilibrium was *p* = 0.86 in the cases vs. *p* = 0.46 in the controls.

### Bioinformatic and differential gene expression analyses

Regulatory functions were annotated with *rSNPBase*^13^. The summarized expression quantitative trait loci (eQTLs) of rs10947540 were obtained from HaploReg v4.1^14^.

Allele-dependent gene expression was determined by the combined use of the ArrayExpress Archive database (http://www.ebi.ac.uk/arrayexpress) and Ensembl (http://www.ensembl.org). Differential gene expression data for *ZNF76*, *DEF6*, and *TAF11* was derived from both our in-house test and the E-GEOD-50772 project from the ArrayExpress Archive database^15^. The E-GEOD-50772 project was conducted with the A-AFFY-44-Affymetrix GeneChip Human Genome U133 Plus 2.0 with RNA from peripheral blood mononuclear cells (PBMCs)^15^.

Our expression analysis was performed with RNA from whole blood. The cDNA library was prepared according to the previously published protocol^16^ and sequenced with PE150 (Illumina, San Diego, California, USA). Clean reads were obtained after filtering out reads containing poly-N, sequencing adapters, and low-quality reads. The remaining clean reads were mapped to the reference genome using Hisat2 software. The expression levels were assessed based on the FPKM (Fragments Per Kilobase of transcript sequence per Millions base pairs sequenced) value.

### Statistical analysis

Hardy–Weinberg equilibrium (HWE) among controls was assessed by the goodness-of-fit χ2 test. Allelic association analyses of SLE patients and healthy controls were performed using the chi-square test. The combined result for meta-analysis was from Cochran–Mantel–Haenszel statistics. Genotype-clinical phenotype association analysis was conducted using the chi-square test for categorical variables and Student’s t-test for continuous variables. Spearman’s coefficient was calculated to determine correlations in allele-dependent gene expression analysis. The differences in *ZNF76*, *DEF6*, and *TAF11* expression between SLE patients and healthy controls were tested using Student's t-test. Statistical analyses were implemented with SPSS 13.0 software. All results with a two-tailed *p* < 0.05 were considered statistically significant.

### Ethics approval

The study was approved by the Medical Ethics Committee of Zhengzhou University First Hospital (2019-KY-134).

### Consent to participate

The informed consent was obtained from all participants and/or their legal guardians.

## Result

### Association of ZNF76 gene polymorphisms with susceptibility to SLE

*ZNF76* rs10947540 was selected as the tag SNP, and the association with SLE was successfully replicated (*p* = 1.60 × 10^−2^, OR 1.19, 95% CI 1.03–1.37) in a larger cohort with 1003 SLE patients and 815 healthy controls (Table 1). After meta-analysis of both the GWAS cohort from the GWAS and our replication cohort, the association between rs10947540 and SLE was pronounced (*P*_*meta*_ = 9.62 × 10^−6^, OR 1.29, 95% CI 1.15–1.44).

### Demographics of SLE patients with three rs10947540 genotypes

Nine hundred ninety-eight SLE patients were successfully genotyped for *ZNF76* rs10947540 and enrolled for clinical association analysis (Table 2). There were trends toward higher incidences of malar rash, discoid rash, photosensitivity, arthritis, serositis, haematological disorder, anti-dsDNA antibodies, and anti-Sm antibodies (without reaching statistical significance) in patients with the risk C allele than in the other patients. Notably, the SLE patients carrying the risk C allele showed significantly higher levels of serum creatinine (Scr) (CC + CT vs. TT, *p* = 9.94 × 10^−4^).

**Table 2:** Prevalence of SLE clinical phenotypes between *ZNF76* rs10947540 polymorphisms in the replication cohort.

Clinical Phenotypes	CC + CT (n = 557)	TT (n = 441)	*p*-value
Onset age (years, mean ± SD)	31 ± 13	32 ± 13	0.140
Gender (male, %)	25 (5.7)	46 (8.3)	0.114
Malar rash (+ , %)	142 (25.5)	109 (24.7)	0.779
Discoid rash (+ , %)	5 (0.9)	2 (0.5)	0.404
Photosensitivity (+ , %)	27 (4.8)	15 (3.4)	0.259
Oral ulcers (+ , %)	40 (7.2)	32 (7.3)	0.964
Arthritis (+ , %)	160 (28.7)	118 (26.8)	0.491
Serositis (+ , %)	53 (9.5)	30 (6.8)	0.123
Renal disorder Scr (μmol/L, mean ± SD)	56 (48–71)	54 (46–64)	9.94 × 10^−4^ (4.29 × 10^−3a^)
24 h UTP (grams, mean ± SD)	2.8 ± 9.1	2.1 ± 2.8	0.363
Neurological disorder (+ , %)	17 (3.1)	18 (4.1)	0.380
Hematological disorder (+ , %)	308 (56.3)	233 (54.8)	0.644
Immunological disorder Anti-dsDNA antibodies (+ , %)	324 (64)	245 (62)	0.536
Anti-Sm antibodies (+ , %)	83 (19.4)	49 (15.1)	0.119
SLEDAI (mean ± SD)	4 ± 4.0	4 ± 4.2	0.741

*Scr* serum creatinine, *24 h UTP* 24-h urinary protein, *SLEDAI* systemic lupus erythematosus disease activity index.

^a^The *p*-value of Scr after adjusted for sex and age.

### Bioinformatic analysis

*ZNF76* rs10947540 was predicted to be a potential regulatory SNP by *rSNPBase*^13^. Experimentally supported regulatory elements from ENCODE and other data resources showed that *ZNF76* rs10947540 was in LD with other regulatory SNPs (r^2^ > 0.8) and had potential distal regulation, RNA-binding protein-mediated regulation, and an eQTL effect. Table 3 shows that rs10947540 was correlated with the expression of *TCP11*, *SCUBE3*, *DEF6*, and *ZNF76* in certain tissues.

**Table 3:** The eQTL effect of rs10947540 in multiple tissues by HaploReg v4.1.

Tissues	Correlated genes	*P*-value
Adipose Visceral Omentum	*TCP11*	1.52 × 10^−6^ ^17^
Cells Transformed fibroblasts	*SCUBE3*	1.78 × 10^−5^ ^17^
Nerve Tibial	*DEF6*	2.61 × 10^−8^ ^17^
Skin Sun Exposed Lower leg	*TCP11*	2.05 × 10^−7^ ^17^
Whole Blood	*DEF6*	1.95 × 10^−21^ ^17^
Whole Blood	*ZNF76*	5.16 × 10^−9^ ^17^
Lymphoblastoid EUR exonlevel	*ENSG00000023892.8 35287301 35287467*	1.42 × 10^−7^ ^18^
Lymphoblastoid EUR exonlevel	*ENSG00000023892.8 35288964 35289548*	8.14 × 10^−8^ ^18^
Lymphoblastoid EUR genelevel	*DEF6*	2.12 × 10^−6^ ^18^
Whole Blood	*DEF6*	2.10 × 10^−132^ ^19^
Whole Blood	*ZNF76*	2.45 × 10^−9^ ^19^
Prepouch ileum	*ZNF76*	1.79 × 10^−6^ ^20^

**Figure 2:**
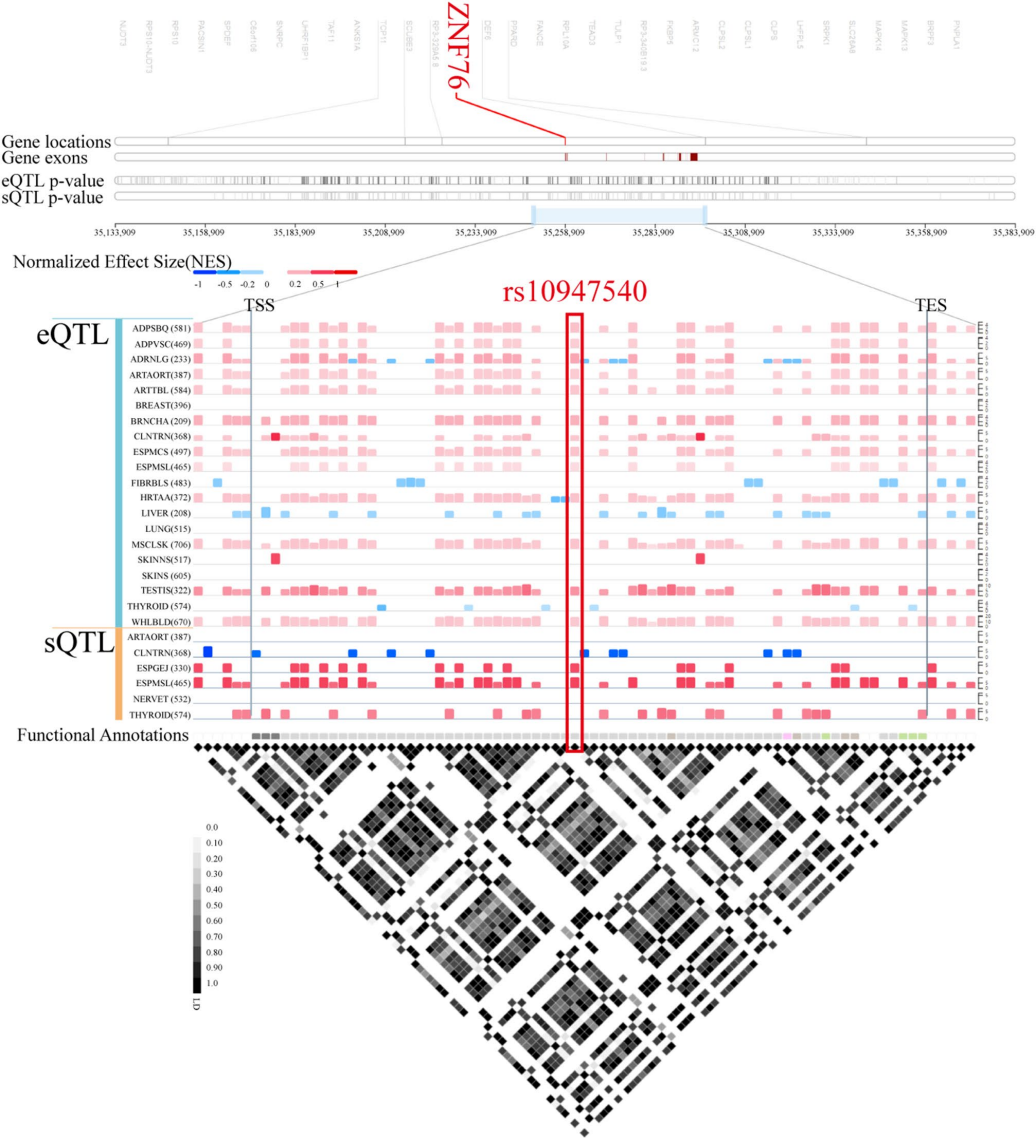
*ZNF76* rs10947540 is an QTL locus. The quantitative trait locus in *ZNF76* were generated by GTEx Locus Browser. *ZNF76* rs10947540 was had eQTL and sQTL effects in multiple tissues.

**Figure 3:**
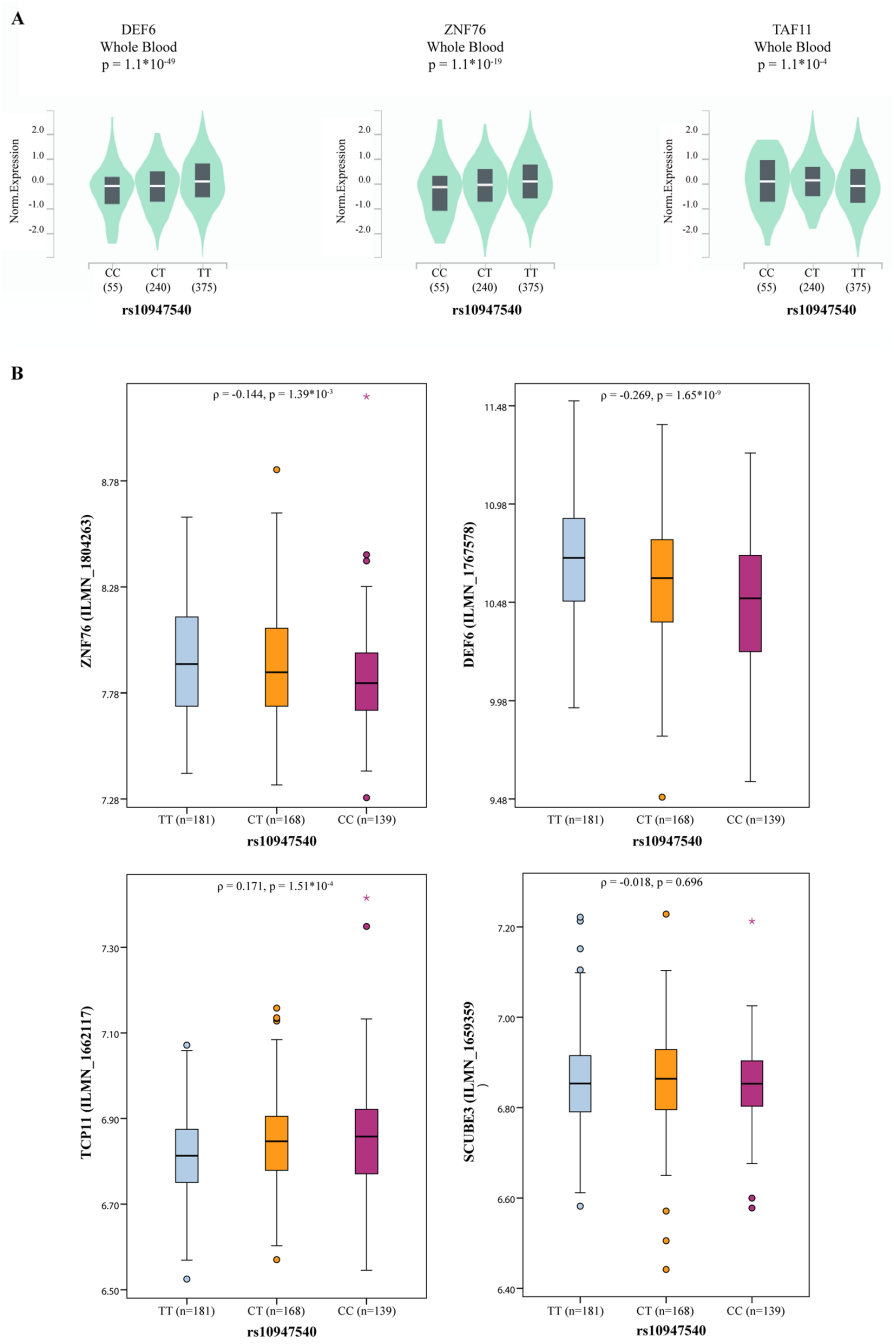
The integrated expression and genotypic analysis of *ZNF76* rs10947540. (**A**) The violin plot for expression of *DEF6*, *ZNF76*, and *TAF11* in whole blood were obtained from GTEx database. (**B**) The correlation between *ZNF76*, *DEF6*, *TCP11*, and *SCUBE3* and rs10947540 genotypes were adopted in E-MTAB-264 and the significances were tested by spearman's correlation coefficient. The boxplot was generated via boxplot in SPSS.

The GTEx Portal provides comprehensive tissue-specific gene expression and regulation data. We inferred from GTEx that rs10947540 is an eQTL (Fig. 2) associated with the expression of 7 genes (*DEF6*, *ZNF76*, *PPARD*, *SCUBE3*, *RPL10A*, *TCP11*, and *TAF11*) in 27 tissues (supplementary Table 2). Individuals carrying the risk C allele had lower expression of *DEF6* (*p* = 1.1 × 10^−49^) and *ZNF76* (*p* = 1.1 × 10^−19^) in whole blood samples (Fig. 3A).

Furthermore, we validated the eQTL effects in 488 individuals from HapMap projections. Individuals carrying the homozygous CC risk allele showed lower levels of *ZNF76* and *DEF6* expression and higher levels of *TCP11* expression than other patients (Fig. 3B). There was no correlation between the *SCUBE3* expression level and rs10947540 genotypes, which might be due to tissue-specific expression.

### Reduced level of ZNF76 was observed in SLE

**Figure 4:**
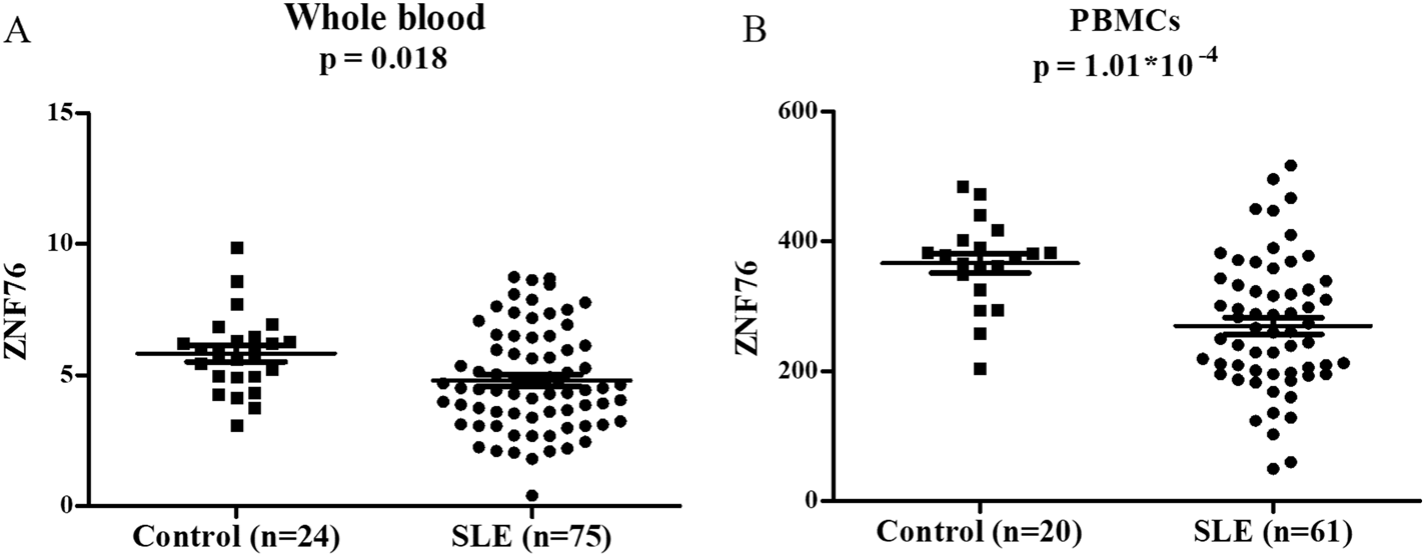
The mRNA expression of *ZNF76* was decreased in SLE patients comparing with controls. The expression levels of *ZNF76* were compared between SLE patients with healthy controls in our cohort shown as FPKM (Fragments Per Kilobase of transcript sequence per Millions base pairs sequenced) (**A**) and E-GEOD-50772 project shown as expression values (**B**). The differences of *ZNF76* expression between SLE patients and healthy controls were calculated by Student's t-test. Abbreviation: PBMC, peripheral blood mononuclear cells; FPKM, fragments per kilobase of transcript sequence per millions base pairs sequenced.

Considering the eQTL effect of rs10947540, we recruited 75 SLE patients and 24 healthy controls to ascertain whether *ZNF76* is differentially expressed. In accordance with the association of the risk C allele with lower levels of gene expression, our expression data for whole blood showed that lower levels of *ZNF76* expression were observed in the patients with SLE (Fig. 4A). Moreover, mRNA expression data from the E-GEOD-50772 project were consistent with our finding that the *ZNF76* expression in peripheral blood mononuclear cells from 61 SLE patients was significantly lower than that in PBMCs from 20 healthy controls (Fig. 4B).

Associated with rs10947540 in whole blood, we perform additional gene expression for *DEF6* and *TAF11*. The expression of TAF11 were significantly lower in SLE patients comparing with healthy controls both in our cohort and E-GEOD-50772 project (Supplement Fig. 1). However, there were no difference in DEF6 expression between SLE patients and healthy controls (Supplement Fig. 1).

## Discussion

To determine whether SNPs in the *ZNF76* gene predispose patients to SLE, we conducted a genetic replication of a previous GWAS genetic association result in an independent Chinese Han population. Our results showed that rs10947540 in *ZNF76* predisposed patients to susceptibility to SLE. In the stratified analysis, we found that patients carrying the risk C allele (CC + CT genotypes) had a more evident risk and higher Scr levels.

*ZNF76*, a novel transcriptional repressor targeting TATA-binding protein, has a strong inhibitory effect on p53 in various cell lines, including the HeLa, U2OS, MCF-7, and H1299 cell lines^9^. Expression data from both our laboratory and the ArrayExpress Archive database demonstrated that the expression of *ZNF76* was lower in patients with SLE than in healthy controls. P53 might be deregulated by the reduced expression of *ZNF76*. The presence of autoantibodies, increased cell apoptosis, and overactivation of type I IFN signalling pathways are prominent characteristics of SLE. In patients with SLE, elevated levels of p53 are detected in fibroblasts, bone marrow-derived mesenchymal stem cells, peripheral blood mononuclear cells, and renal tissues; they are also found in the skin of discoid lupus erythematosus patients^21–25^. Researchers have reported that increased apoptosis is associated with p53 upregulation in p21^-/-^ lupus mice^26^. Moreover, p53 activation in SLE patients may stimulate type I IFN activity, promoting innate immune signalling directly^27,28^. The production of autoantibodies requires p53 in B6/lpr lupus mice^29^. We hypothesized that the reduced expression of *ZNF76* promoted the pathogenesis of SLE, which might be due to deregulation of p53.

DEF6 is required for maintaining T cell effector functions and lymphocyte homeostasis and preventing systemic autoimmunity^30^. Mice deficient in DEF6 can spontaneously develop a lupus-like syndrome with increased levels of autoantibodies and glomerulonephritis^30^. The bioinformatic analysis revealed that *ZNF76* rs10947540 was annotated as an eQTL associated with the expression of *DEF6*. Furthermore, genotyping and expression data from a HapMap population confirmed that the risk allele of rs10947540 was correlated with decreased expression of *DEF6*. We could not rule out the possibility that rs10947540 might promote the development of SLE by affecting *DEF6* expression (supplementary Fig. 1). *ZNF76* functions as a transcriptional repressor. Whether the reduced expression of *DEF6* was due to the regulation of *ZNF76* remains to be elucidated.

The risk allele was associated with decreased *ZNF76* expression levels according to data for individuals from the GTEx database and the expression data of HapMap3 projections. However, the data from the GTEx database and the expression data of HapMap3 projections were from healthy individuals. Thus, a limitation of this study was that we lacked data from SLE patients to explore the association between the risk allele and *ZNF76* expression.

Our present study demonstrates that the rs10947540 polymorphism of the *ZNF76* gene is a possible susceptibility factor associated with SLE susceptibility. The mechanism underlying the association between *ZNF76* and the pathogenesis of SLE still requires further investigation.

## Supplementary Information

Supplementary Information 1.

Supplementary Information 2.

## Data Availability

The data that support the findings of this study are available from the corresponding author upon reasonable request.

## References

[1] Petukhova L (2010). Genome-wide association study in alopecia areata implicates both innate and adaptive immunity. Nature.

[2] Wu AC (2014). Inhaled corticosteroid treatment modulates ZNF432 gene variant's effect on bronchodilator response in asthmatics. J. Allergy Clin. Immunol..

[3] Xie G (2013). Identification of the NF-κB activating protein-like locus as a risk locus for rheumatoid arthritis. Ann. Rheum. Dis..

[4] Haritunians T (2011). Variants in ZNF365 isoform D are associated with Crohn's disease. Gut.

[5] Li L (2018). Polymorphisms of IKZF3 gene and autoimmune thyroid diseases: associated with graves' disease but not with hashimoto's thyroiditis. Cell. Physiol. Biochem. Int. J. Exp. Cell. Physiol. Biochem. Pharmacol..

[6] Lessard CJ (2012). Identification of IRF8, TMEM39A, and IKZF3-ZPBP2 as susceptibility loci for systemic lupus erythematosus in a large-scale multiracial replication study. Am. J. Hum. Genet..

[7] Cai X (2014). Association between polymorphisms of the IKZF3 gene and systemic lupus erythematosus in a Chinese Han population. PLoS ONE.

[8] Zhang YM (2017). Association of the IKZF1 5' UTR variant rs1456896 with lupus nephritis in a northern Han Chinese population. Scand. J. Rheumatol..

[9] Zheng G, Yang Y-C (2004). ZNF76, a novel transcriptional repressor targeting TATA-binding protein, is modulated by sumoylation. J. Biol. Chem..

[10] Yang F (2020). Bioinformatics identification of key candidate genes and pathways associated with systemic lupus erythematosus. Clin. Rheumatol..

[11] Hochberg MC (1997). Updating the American College of Rheumatology revised criteria for the classification of systemic lupus erythematosus. Arthritis Rheum..

[12] Sun C (2016). High-density genotyping of immune-related loci identifies new SLE risk variants in individuals with Asian ancestry. Nat. Genet..

[13] Guo L, Du Y, Qu S, Wang J (2016). rVarBase: an updated database for regulatory features of human variants. Nucl. Acids Res..

[14] Ward LD, Kellis M (2012). HaploReg: a resource for exploring chromatin states, conservation, and regulatory motif alterations within sets of genetically linked variants. Nucl. Acids Res..

[15] Kennedy WP (2015). Association of the interferon signature metric with serological disease manifestations but not global activity scores in multiple cohorts of patients with SLE. Lupus Sci. Med..

[16] Parkhomchuk D (2009). Transcriptome analysis by strand-specific sequencing of complementary DNA. Nucl. Acids Res..

[17] Ardlie KG (2015). Human genomics: the genotype-tissue expression (GTEx) pilot analysis: multitissue gene regulation in humans. Science.

[18] Lappalainen T (2013). Transcriptome and genome sequencing uncovers functional variation in humans. Nature.

[19] Westra HJ (2013). Systematic identification of trans eQTLs as putative drivers of known disease associations. Nat. Genet..

[20] Kabakchiev B, Silverberg MS (2013). Expression quantitative trait loci analysis identifies associations between genotype and gene expression in human intestine. Gastroenterology.

[21] Günther C (2015). Defective removal of ribonucleotides from DNA promotes systemic autoimmunity. J. Clin. Investig..

[22] Gao L (2017). Bone marrow-derived mesenchymal stem cells from patients with systemic lupus erythematosus have a senescence-associated secretory phenotype mediated by a mitochondrial antiviral signaling protein-interferon-β feedback loop. Arthritis Rheumatol..

[23] Zamolo G (2005). Expression of p53 and apoptosis in discoid lupus erythematosus. Croat. Med. J..

[24] El-Sayed ZA, Farag DH, Eissa S (2003). Tumor suppressor protein p53 and anti-p53 autoantibodies in pediatric rheumatological diseases. Pediatr. Allergy Immunol..

[25] Wang JS, Tseng HH, Shih DF, Jou HS, Ger LP (1997). Expression of inducible nitric oxide synthase and apoptosis in human lupus nephritis. Nephron.

[26] Lawson BR (2004). Deficiency of the cyclin kinase inhibitor p21(WAF-1/CIP-1) promotes apoptosis of activated/memory T cells and inhibits spontaneous systemic autoimmunity. J. Exp. Med..

[27] Brzostek-Racine S, Gordon C, Van Scoy S, Reich NC (2011). The DNA damage response induces IFN. J. Immunol..

[28] Muñoz-Fontela C (2008). Transcriptional role of p53 in interferon-mediated antiviral immunity. J. Exp. Med..

[29] Kuan AP, Cohen PL (2005). p53 is required for spontaneous autoantibody production in B6/lpr lupus mice. Eur. J. Immunol..

[30] Fanzo JC (2006). Loss of IRF-4-binding protein leads to the spontaneous development of systemic autoimmunity. J. Clin. Invest..

