# Supplementary material for: The ZNF76 rs10947540 polymorphism associated with systemic lupus erythematosus risk in Chinese populations
Source: Sci Rep. 2021 Mar 4;11:5186. doi: 10.1038/s41598-021-84236-3 (PMC7933287; doi:10.1038/s41598-021-84236-3)
Supplement: Supplementary file 1 — Supplementary Information 1. [file 41598_2021_84236_MOESM1_ESM.docx]

**The *ZNF76* rs10947540 polymorphism associated with systemic lupus erythematosus risk in Chinese populations**

Yuan-yuan Qi^1#^, Yan Cui^1#^, Hui Lang^2^, Ya-ling Zhai^1^, Xiao-xue Zhang^1^, Xiao-yang Wang^1^, Xin-ran Liu^1^, Ya-fei Zhao^1^, Xiang-hui Ning^3^, Zhan-Zheng Zhao^1*^

**AUTHORS’ INSTITUTION AND AFFILIATION**

1. Nephrology Hospital, the First Affiliated Hospital of Zhengzhou University, Zhengzhou University, Henan 4500052, China;
2. School of Pharmaceutical Sciences, Zhengzhou University, Zhengzhou 450001, PR China;
3. Department of Urology, the First Affiliated Hospital of Zhengzhou University, Henan 4500052, China.

^#^: These authors contribute equally to this work.

CORRESPONDING AUTHOR

Dr. Zhan-zheng Zhao, MD & PhD;

Email: zhanzhengzhao@zzu.edu.cn

Nephrology Hospital, the First Affiliated Hospital of Zhengzhou University,

Institute of Nephrology, Zhengzhou University

No.1, Jianshe Road, Erqi District

Zhengzhou 4500052, P.R China

**KEY WORDS**

Systemic lupus erythematosus, Single nucleotide polymorphisms, ZNF76*,* rs10947540

| Supplementary table1. Association results of SNPs in *ZNF76* and SLE susceptibility. | | | | | | |
| --- | --- | --- | --- | --- | --- | --- |
| SNP | Chr. | Pos (hg19) | Minor allele | MAF (Case/Control, %) | P-value | OR(95%CI) |
| rs4713844 | 6 | 35227832 | T | 2.1/1.6 | 0.40 | 1.33(0.69-2.56) |
| rs9296144 | 6 | 35228215 | G | 38.5/29.6 | 3.4*10^-5^ | 1.49(1.23-1.79) |
| rs10080361 | 6 | 35228243 | A | 39/29.9 | 2.4*10^-5^ | 1.50(1.24-1.80) |
| rs4713845 | 6 | 35228387 | C | 39.5/30.2 | 1.6*10^-5^ | 1.51(1.25-1.82) |
| rs4713846 | 6 | 35228828 | A | 1.2/0.6 | 0.15 | 2.03(0.76-5.43) |
| rs1888825 | 6 | 35230540 | G | 39.5/30.2 | 1.6*10^-5^ | 1.51(1.25-1.82) |
| rs9462065 | 6 | 35231168 | T | 40.5/32 | 7.8*10^-5^ | 1.45(1.21-1.75) |
| rs975496 | 6 | 35233443 | A | 39/29.9 | 2.4*10^-5^ | 1.50(1.24-1.80) |
| rs2267657 | 6 | 35235858 | G | 40.6/32 | 6.5*10^-5^ | 1.46(1.21-1.75) |
| rs2267658 | 6 | 35235871 | G | 39.1/29.9 | 1.9*10^-5^ | 1.50(1.25-1.81) |
| rs6934083 | 6 | 35237153 | T | 0.6/0.5 | 0.76 | 1.21(0.37-3.97) |
| rs6941471 | 6 | 35238626 | G | 40.2/31.6 | 1*10^-4^ | 1.46(1.21-1.76) |
| rs2267660 | 6 | 35242123 | G | 36.9/28.2 | 3.5*10^-5^ | 1.49(1.23-1.80) |
| rs2267661 | 6 | 35242181 | T | 36.9/28.2 | 3.5*10^-5^ | 1.49(1.23-1.80) |
| rs10947540 | 6 | 35243257 | C | 39.6/30.2 | 1.31*10^-5^ | 1.51(1.25-1.82) |
| rs6902523 | 6 | 35244531 | G | 39.1/29.9 | 1.9*10^-5^ | 1.50(1.25-1.81) |
| rs6457812 | 6 | 35245120 | C | 40.6/32 | 6.5*10^-5^ | 1.46(1.21-1.75) |
| rs10484578 | 6 | 35246319 | G | 33.5/40.8 | 8.1*10^-4^ | 0.73(0.61-0.88) |
| rs6919534 | 6 | 35246903 | G | 2.1/1.6 | 0.40 | 1.33(0.69-2.56) |
| rs9394289 | 6 | 35247904 | C | 39.6/30.2 | 1.31*10^-5^ | 1.51(1.25-1.82) |
| rs7772212 | 6 | 35249908 | C | 39.1/29.9 | 1.9*10^-5^ | 1.50(1.25-1.81) |
| rs2267662 | 6 | 35250440 | C | 39.1/29.9 | 1.9*10^-5^ | 1.50(1.25-1.81) |
| rs2267663 | 6 | 35250682 | C | 39.6/30.2 | 1.31*10^-5^ | 1.51(1.25-1.82) |
| rs12203818 | 6 | 35251317 | A | 3/4.1 | 0.19 | 0.72(0.44-1.17) |
| rs3798342 | 6 | 35251494 | A | 36.9/28.3 | 4.4*10^-5^ | 1.48(1.23-1.79) |
| rs114389319 | 6 | 35254557 | A | 0.5/0.6 | 0.77 | 0.84(0.25-2.75) |
| rs729925 | 6 | 35256934 | C | 1.9/1.5 | 0.48 | 1.28(0.65-2.53) |
| rs763157 | 6 | 35258539 | C | 1.9/1.5 | 0.48 | 1.28(0.65-2.53) |
| rs1894650 | 6 | 35259261 | C | 39.6/30.2 | 1.31*10^-5^ | 1.51(1.25-1.82) |
| rs33959228 | 6 | 35259397 | T | 0.4/0.6 | 0.53 | 0.67(0.19-2.38) |
| rs9366883 | 6 | 35259646 | G | 39.6/30.2 | 1.31*10^-5^ | 1.51(1.25-1.82) |
| rs1557568 | 6 | 35260530 | C | 40.6/32 | 6.5*10^-5^ | 1.46(1.21-1.75) |
| rs4713848 | 6 | 35260615 | C | 36.9/28.3 | 4.4*10^-5^ | 1.48(1.23-1.79) |
| rs4711413 | 6 | 35260858 | A | 36.9/28.3 | 4.4*10^-5^ | 1.48(1.23-1.79) |
| rs965272 | 6 | 35261990 | C | 40.6/32 | 6.5*10^-5^ | 1.46(1.21-1.75) |
| rs1194 | 6 | 35263555 | A | 38.9/29.8 | 2.3*10^-5^ | 1.50(1.24-1.81) |
| rs8205 | 6 | 35263677 | T | 40.4/31.9 | 7.8*10^-5^ | 1.45(1.21-1.75) |

| Supplementary table2. eQTLs of rs1888822 by GTEx. | | | |  |
| --- | --- | --- | --- | --- |
| Gencode Id | Gene Symbol | P-Value | Tissue | |
| ENSG00000023892.10 | *DEF6* | 1.10E-49 | Whole Blood | |
| ENSG00000065029.14 | *ZNF76* | 1.10E-19 | Whole Blood | |
| ENSG00000023892.10 | *DEF6* | 5.70E-15 | Nerve - Tibial | |
| ENSG00000124678.17 | *TCP11* | 2.70E-11 | Adipose - Subcutaneous | |
| ENSG00000124678.17 | *TCP11* | 3.20E-11 | Skin - Sun Exposed (Lower leg) | |
| ENSG00000146197.8 | *SCUBE3* | 4.10E-11 | Cells - Cultured fibroblasts | |
| ENSG00000065029.14 | *ZNF76* | 3.90E-10 | Testis | |
| ENSG00000198755.10 | *RPL10A* | 5.50E-10 | Artery - Tibial | |
| ENSG00000065029.14 | *ZNF76* | 2.00E-09 | Adrenal Gland | |
| ENSG00000065029.14 | *ZNF76* | 2.40E-09 | Muscle - Skeletal | |
| ENSG00000023892.10 | *DEF6* | 3.50E-09 | Pituitary | |
| ENSG00000198755.10 | *RPL10A* | 3.90E-09 | Esophagus - Muscularis | |
| ENSG00000124678.17 | *TCP11* | 1.20E-08 | Skin - Not Sun Exposed (Suprapubic) | |
| ENSG00000023892.10 | *DEF6* | 1.40E-08 | Heart - Left Ventricle | |
| ENSG00000023892.10 | *DEF6* | 5.20E-08 | Lung | |
| ENSG00000023892.10 | *DEF6* | 6.00E-08 | Ovary | |
| ENSG00000065029.14 | *ZNF76* | 7.90E-08 | Artery - Tibial | |
| ENSG00000065029.14 | *ZNF76* | 2.20E-07 | Esophagus - Mucosa | |
| ENSG00000023892.10 | *DEF6* | 2.40E-07 | Esophagus - Gastroesophageal Junction | |
| ENSG00000065029.14 | *ZNF76* | 3.10E-07 | Artery - Aorta | |
| ENSG00000065029.14 | *ZNF76* | 1.2E-06 | Heart - Atrial Appendage | |
| ENSG00000124678.17 | *TCP11* | 2.2E-06 | Esophagus - Mucosa | |
| ENSG00000023892.10 | *DEF6* | 3.6E-06 | Cells - Cultured fibroblasts | |
| ENSG00000198755.10 | *RPL10A* | 3.7E-06 | Adipose - Visceral (Omentum) | |
| ENSG00000198755.10 | *RPL10A* | 3.7E-06 | Colon - Sigmoid | |
| ENSG00000124678.17 | *TCP11* | 3.8E-06 | Testis | |
| ENSG00000065029.14 | *ZNF76* | 4.8E-06 | Adipose - Subcutaneous | |
| ENSG00000124678.17 | *TCP11* | 6.3E-06 | Adipose - Visceral (Omentum) | |
| ENSG00000198755.10 | *RPL10A* | 6.9E-06 | Muscle - Skeletal | |
| ENSG00000198755.10 | *RPL10A* | 7.2E-06 | Nerve - Tibial | |
| ENSG00000124678.17 | *TCP11* | 8.4E-06 | Pituitary | |
| ENSG00000023892.10 | *DEF6* | 0.000011 | Adipose - Visceral (Omentum) | |
| ENSG00000124678.17 | *TCP11* | 0.000011 | Artery - Tibial | |
| ENSG00000023892.10 | *DEF6* | 0.000011 | Cells - EBV-transformed lymphocytes | |
| ENSG00000023892.10 | *DEF6* | 0.000016 | Artery - Tibial | |
| ENSG00000065029.14 | *ZNF76* | 0.000016 | Brain - Cerebellum | |
| ENSG00000124678.17 | *TCP11* | 0.000018 | Heart - Atrial Appendage | |
| ENSG00000112033.13 | *PPARD* | 0.000024 | Skin - Not Sun Exposed (Suprapubic) | |
| ENSG00000198755.10 | *RPL10A* | 0.000031 | Thyroid | |
| ENSG00000023892.10 | *DEF6* | 0.000042 | Thyroid | |
| ENSG00000124678.17 | *TCP11* | 0.000043 | Breast - Mammary Tissue | |
| ENSG00000023892.10 | *DEF6* | 0.000046 | Esophagus - Muscularis | |
| ENSG00000023892.10 | *DEF6* | 0.000049 | Adipose - Subcutaneous | |
| ENSG00000023892.10 | *DEF6* | 0.000072 | Brain - Cerebellum | |
| ENSG00000023892.10 | *DEF6* | 0.000083 | Brain - Caudate (basal ganglia) | |
| ENSG00000064995.16 | *TAF11* | 0.00011 | Whole Blood | |
| ENSG00000065029.14 | *ZNF76* | 0.00012 | Esophagus - Muscularis | |
| ENSG00000065029.14 | *ZNF76* | 0.00013 | Adipose - Visceral (Omentum) | |
| ENSG00000065029.14 | *ZNF76* | 0.00016 | Colon - Transverse | |
| ENSG00000198755.10 | *RPL10A* | 0.00016 | Artery - Aorta | |

Supplementary Figure 1. The mRNA expression of *DEF6* and *TAF11* in SLE patients comparing with controls.

The expression of *DEF6* and *TAF11* were compared between SLE patients with healthy controls in our cohort shown as FPKM (Fragments Per Kilobase of transcript sequence per Millions base pairs sequenced) (A) and E-GEOD-50772 project shown as expression values (B). The differences of *ZNF76* expression between SLE patients and healthy controls were calculated by Student's t-test.
